# Flowering and fruiting synchronization, pollen number, floral visitors and reproductive success of *Paubrasilia echinata* (brazilwood; Leguminosae) in tropical urban ecosystem in comparison to Atlantic forest remnant: A dataset description

**DOI:** 10.1016/j.dib.2019.104177

**Published:** 2019-06-22

**Authors:** Willams Oliveira, Jéssica Luiza Souza e Silva, Marcela Tomaz Pontes de Oliveira, Oswaldo Cruz-Neto, Luanda Augusta Pinheiro da Silva, Laís Angélica Borges, Mellissa Sousa Sobrinho, Ariadna Valentina Lopes

**Affiliations:** a Departamento de Botânica, Centro de Biociências (CB), Universidade Federal de Pernambuco (UFPE), Pernambuco, 50372-970, Brazil; b Departamento de Ciências Biológicas, Centro de Ciências Agrárias, Universidade Federal da Paraíba (UFPB), 58397-000, Brazil; c Universidade Federal do Amapá (UNIFAP), Campus Mazagão, 68940-000, Brazil

**Keywords:** Reproductive phenology, Pollination, Bee, Seed set, Urbanization, Tropical green spaces

## Abstract

In this article, we supply raw data on the reproductive biology and frequency of pollinators of *Paubrasilia echinata*, a threatened tree, endemic to the Brazilian Atlantic forest, which is largely used in Brazilian urban areas (e.g. avenues, parks and squares) due to its ornamental potential. Specifically, we share data on the reproductive phenology, pollen/flower, floral visitors and seed set of *P. echinata* in urban and natural ecosystems. This dataset article is related to the original research article "**Reduced reproductive success of the endangered tree brazilwood (*Paubrasilia echinata*, Leguminosae) in urban ecosystem compared to Atlantic forest remnant: lessons for tropical urban ecology**" (Oliveira et al., 2019). As urbanization is thought to negatively impact the maintenance of plant communities by affecting ecological key interactions, such as pollination, we believe that data as the supplied here are relevant and could support the planning of urban green spaces to maintain viable communities of plants and animals. This is especially valid for tropical urban ecosystems since most of the studies on plant ecology have been developed in temperate regions and there are still several gaps on the knowledge of ecological functions and ecosystems services (e.g. pollination) in urban green areas in the tropics.

**Table:** Specifications table

*Subject area*	*Biology*
*More specific subject area*	*Plant reproductive ecology, pollination ecology, plant reproductive phenology, urban ecology.*
*Type of data*	*Tables, text file, graphs and figures.*
*How data was acquired*	*Binoculars for phenological data; microscopes and manual counter for evaluating reproductive success of P. echinata.*
*Data format*	*Raw and analysed.*
*Experimental features*	*Many components of tree reproduction such as synchronization of reproductive phenology, pollen number/flower, floral visitors and seed set were compared between urban and natural ecosystems.*
*Data source location*	*Urban areas located in Recife Municipality (08°03′15″ S; 34°52′53″ W), and natural area in São Lourenço da Mata Municipality (08°00′13″ S; 35°01′17″ W), Pernambuco State, Brazil.*
*Data accessibility*	*Data is with this article.*
*Related research article*	*Data presented in this brief is related to the study “Reduced reproductive success of the endangered tree brazilwood (Paubrasilia echinata, Leguminosae) in urban ecosystem compared to Atlantic forest remnant: lessons for tropical urban ecology’’**[1]**.*

**Table:** 

**Value of the data** • Synchrony level in flowering and fruiting provide strong evidence about how planted trees in urban green spaces may supply food resource for populations of pollinators and seed dispersers of urban areas and surrounding forest remnants. • Comparisons of the number of pollen grains per flower of a plant species occurring in urban and natural ecosystems may be useful to indirectly access the reproductive potential of the species in novel ecosystems such as the urban green spaces. • Data on the frequency of floral visitors and reproductive success, assessed from seed set, is a reliable measure and may be relevant to understand the effectiveness of pollination of *P. echinata* in urban and natural ecosystems. • These data are important for the planning of tropical urban ecosystems (e.g. parks and squares) and to support strategies for ex-situ conservation of native species such as brazilwood (*Paubrasilia echinata*) in urban green spaces.

## 1 Data

**Fig. 1:**
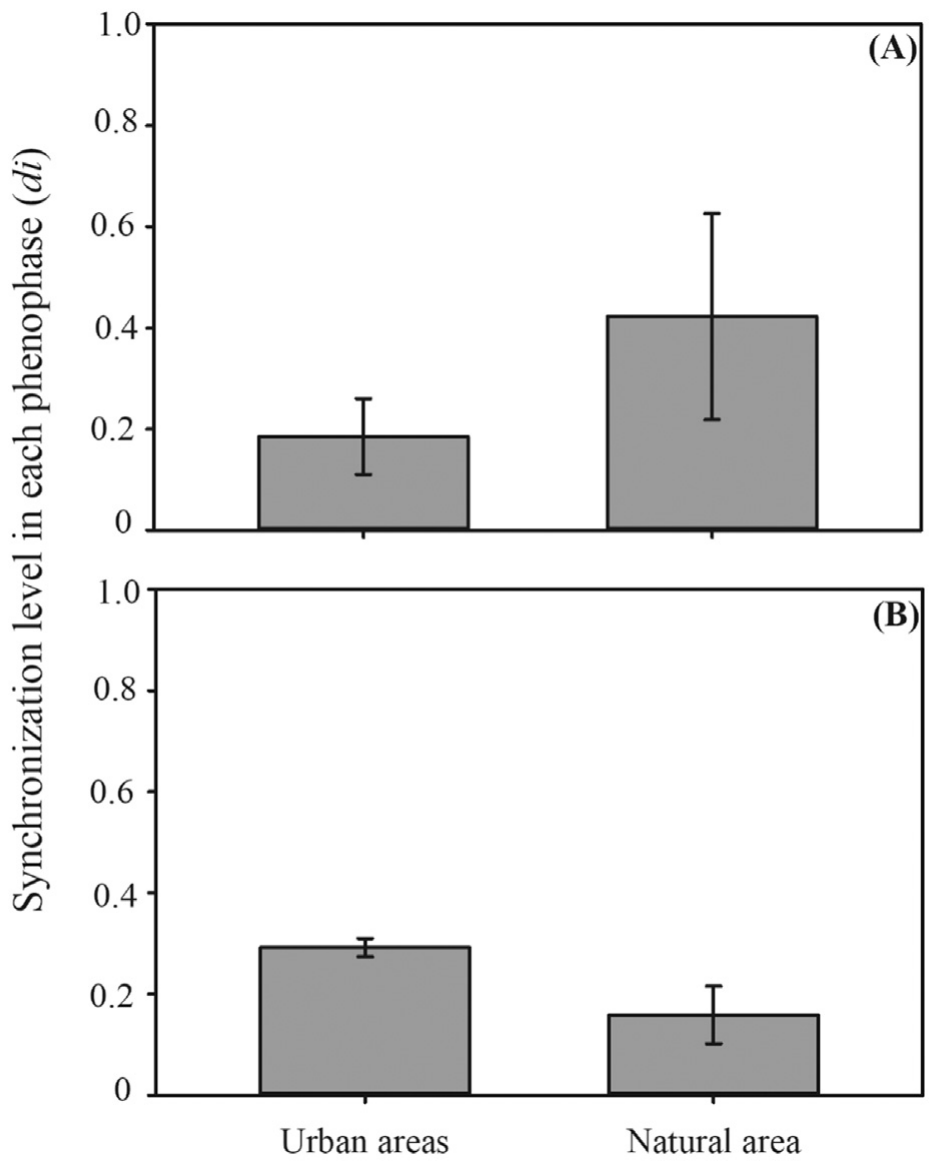
Level of flowering (A) and fruiting (B) synchronization of *Paubrasilia echinata* (Leguminosae) in populations occurring in urban green areas (UA; 20 individuals in 5 urban areas) and natural area (NA; 15 individuals) in Pernambuco State, Brazil. Bars = Level (mean) of flowering and fruiting synchronization in urban and natural populations. Vertical line in each bar represents the standard deviation of the mean. Values between urban and natural areas were significantly different for fruiting synchrony (t = 6.67; p < 0.00001), but not for flowering synchrony (t = −1.33; p = 0.19).

**Fig. 2:**
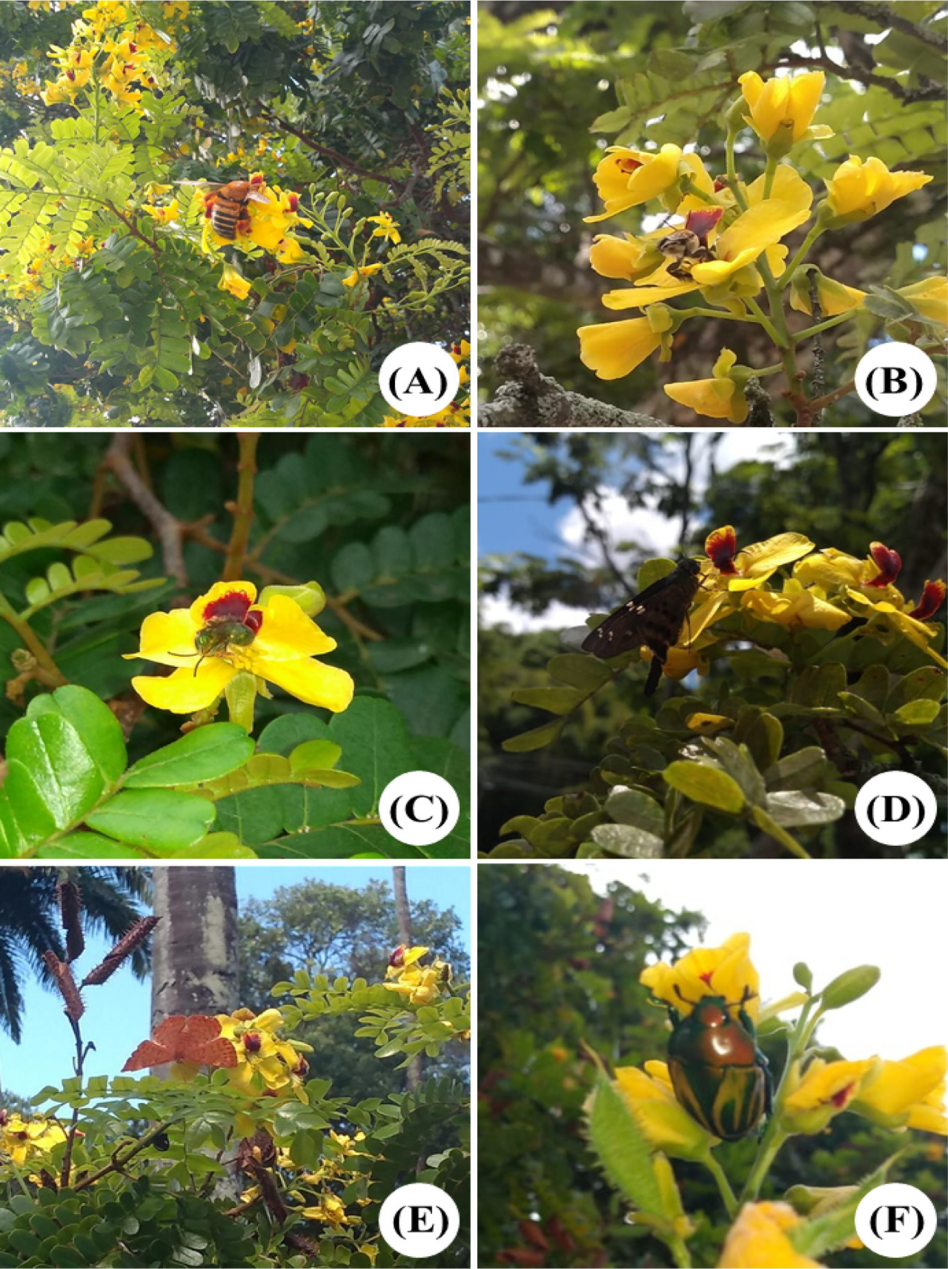
Some of the floral visitors of *Paubrasilia echinata* (Leguminosae) in urban green spaces (A, C, E, F) and natural (B, D) ecosystems in Pernambuco State, Brazil. Effective pollinator: (A) *Xylocopa frontalis* and (B) *Centris aenea*; Occasional pollinators: (C) *Augochloropsis* sp. and (D) *Proteides mercurius*; Nectar thief: (E) Nymphalidae; and Florivore: (F) *Macraspis festiva*.

**Fig. 3:**
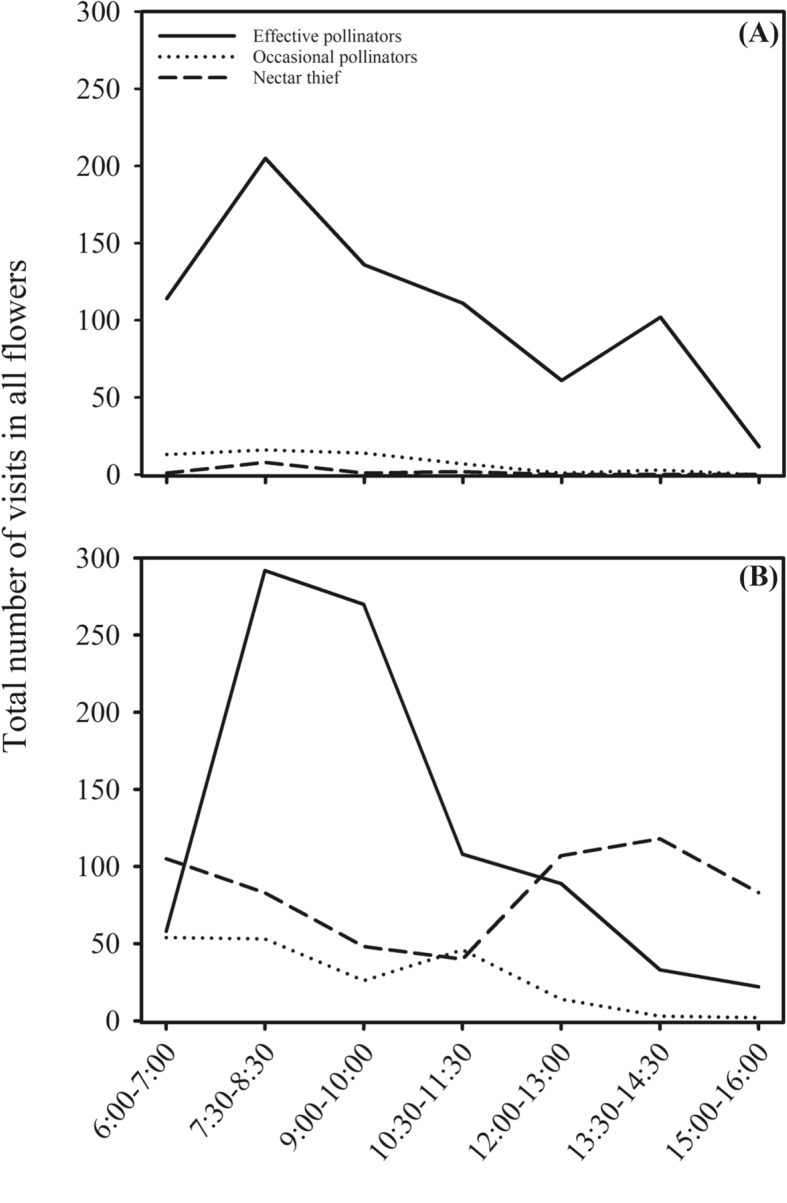
Frequency (mean) of floral visitors in *Paubrasilia echinata* (Leguminosae) in urban (A) and natural ecosystems (B) in Pernambuco State, Brazil (30 hours/ecosystem).

**Fig. 4:**
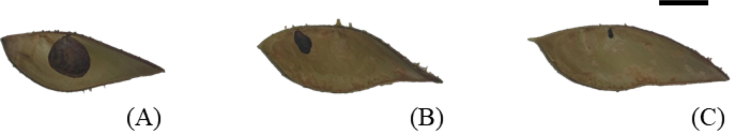
Fully developed seed (A), malformed seed (B) and aborted/unfertilized ovule (C) documented in mature fruits of *Paubrasilia echinata* (Leguminosae) in urban green spaces and also observed in natural area in Pernambuco State, Brazil. (Photos from urban areas) (Scale bar = 20 mm).

Data on the reproductive success of *P. echinata* in urban green spaces and natural forest remnant in the Brazilian northeastern Atlantic forest are shared in this article. Specifically, data on geographical location (Table 1), flowering and fruiting synchrony (Fig. 1), pollen number/flower (Table 2), floral visitors (Fig. 2) and seed set are described. Differences on the number of pollen grains from large and smaller anthers, counted with the use of a manual counter, are explored in urban and natural ecosystems (Table 2). Data on the frequency of floral visitors and their behaviour in both studied ecosystems are also provided (Table 3, Fig. 3). In addition, the description and incidence of intact and malformed seeds and ovule abortion in developed fruits in urban and natural areas are documented in Table 4 and Fig. 4.

**Table 1:** Geographical coordinates of the study sites in tropical urban and natural ecosystems in Pernambuco State, Brazil.

Study sites	Individuals	Latitude	Longitude
*Natural*			
Tapacurá Ecological Station	15	08°02′23″ S	35°11′40″ W
*Urban*			
Parque Treze de Maio	3	08°03′30″ S	34°52′56″ W
Parque da Jaqueira	5	08°02′12″ S	34°54′10″ W
Praça de Casa Forte	2	08°03′24″ S	34°53′53″ W
Praça do Derby	3	08°03′41″ S	34°52′45″ W
Praça da República	7	08°02′09″ S	34°55′09″ W

**Table 2:** Number of pollen grains per anther and per flower of *Paubrasilia echinata* Lam. (Leguminosae) in tropical urban and natural ecosystems in Pernambuco State, Brazil.

Attributes	N	Mean ± SD				Test	P
		Urban		Natural			
Pollen/anther							
Smaller	30	700.91 ± 209.15aA		801,80 ± 210,63aA		t = −1.75^a^	0.0858^a^
Larger	30	903.51 ± 179.55aB		999.08 ± 239.70aB		t = −1.86^a^	0.0677^a^
		**Test**	**P**	**Test**	**P**		
		t = −4.02^b^	0.0001^b^	t = −3.38^c^	0.0001^c^		
Pollen/flower	30	9004.4 ± 2140.3		8022.17 ± 1458.93		t = 9.93^a^	< 0.0001^a^

Values in the same line followed by different lowercase letters were statistically different (p < 0.05); Values of pollen/anther and pollen/flower in a same column followed by different uppercase letters were statistically different (p < 0.05).

a Values for comparison in a same line.

b Comparisons within urban ecosystem.

c Comparisons within natural ecosystem.

**Table 3:** Frequency (mean ± S.D.) of floral visitors in *Paubrasilia echinata* (Leguminosae) in tropical urban and natural ecosystems in Pernambuco State, Brazil (30 h/ecosystem; three individuals/ecosystem).

Visitors behaviour	N (U/N)	Frequency (Mean ± SD)		Test	P
		Urban	Natural		
Effective pollinator	2/5	33.95 ± 8.62	29.41 ± 30.77	t = 1.15	0.26
Occasional pollinator	3/4	0.04 ± 0.21	2.82 ± 3.35	t = −1.27	0.20
Nectar thief	2/2	2.95 ± 3.41	19.41 ± 8.67	t = −3.64	0.0003

**Table 4:** Seed set (fully developed seeds, malformed seeds and aborted ovule) of *Paubrasilia echinata* (Leguminosae) in urban compared to natural areas in Pernambuco State, Brazil.

Seed set	Mean ± SD		Test	P
	Urban	Natural		
Fully developed seed	1.11 ± 0.84	1.63 ± 0.93	t = −4.16	<0.00001
Malformed seed	0.25 ± 0.54	0.14 ± 0.45	t = −1.57	0.11846
Aborted ovule	0.06 ± 0.24	0.11 ± 0.34	t = −1.19	0.23466

## 2 Experimental design, materials and methods

### 2.1 Flowering and fruiting synchrony

The level of synchronization of an individual in relation to all other sampled individuals (20 in urban and 15 in natural; Table 1) (di) was calculated to observe changes in flowering and fruiting synchrony between urban green spaces and natural area. We calculated the (di), which is based on the number of censuses and the intensity in which the analysed phenophase was exhibited for *P. echinata* populations in urban green spaces (Fig. 1). The (di) represents a more accurate measure of the synchrony of a phenophase in relation to other widely used indices, since it considers the intensity [2] and the overlap of the phenophase at the individual or population levels [3]. Trees of each ecosystem were pooled together in all the statistical analyses (i.e. phenological, pollen number, frequency of floral visitors, and reproductive success).

### 2.2 Pollen number

The male component of the reproductive system of brazilwood (*P. echinata*) has two whorls of five stamens each, with large (L) and small (S) anthers. Differences in the amount of pollen grains per anther between trees from urban and natural populations and between L-anther and S-anther were tested. For this, anthers were longitudinally opened under optical microscope, and had all pollen grains removed for counting using a manual counter. The average number of pollen grains per L- and S-anther were obtained from 30 intact flower buds in pre-anthesis, collected from both ecosystems (Table 2).

### 2.3 Frequency of floral visitors

The frequency of floral visitors was observed for about 30 hours in three individuals of *P. echinata* in each ecosystem (urban and natural). We registered the visitors and their approaching behaviours to the flowers. According to the visiting behaviour, floral visitors were classified as: a) effective pollinators, when the visitor contacted both reproductive structures of the flower in a same visit while collecting nectar, in all observed visits; b) occasional pollinator, when the visitor acted as a pollinator but did not contact the flower structures in all visits; c) thief, when the visitor collected nectar without contacting the reproductive structures of the flower (Fig. 2, Fig. 3). To check for differences on the frequency of floral visitors between urban and natural ecosystems we used general linear models (GLM) with Gaussian distribution and “identity” link function (Table 3) [4].

### 2.4 Reproductive success

The female reproductive success was accessed through the average number of seeds per fruit under natural conditions (seed/fruit). To check for differences in seed set per fruit between urban and natural ecosystems, a total of 100 fruits from 10 individuals in both ecosystems were collected and their seeds were counted and classified in (1) fully developed, (2) malformed and (3) aborted/unfertilized ovule according to their development (Table 4 and Fig. 4). The number of seeds in each category was compared by using generalized linear models (GLM) with binomial distribution and “logit” link function (Table 4) [4].
